# Electrochemically Deposited Molecularly Imprinted Polymer-Based Sensors

**DOI:** 10.3390/s22031282

**Published:** 2022-02-08

**Authors:** Simonas Ramanavičius, Inga Morkvėnaitė-Vilkončienė, Urtė Samukaitė-Bubnienė, Vilma Ratautaitė, Ieva Plikusienė, Roman Viter, Arūnas Ramanavičius

**Affiliations:** 1 Department of Electrochemical Material Science, State Research Institute Center for Physical Sciences and Technology (FTMC), Sauletekio av. 3, LT-10257 Vilnius, Lithuania; simonas.ramanavicius@ftmc.lt; 2 Department of Physical Chemistry, Faculty of Chemistry and Geosciences, Institute of Chemistry, Vilnius University, Naugarduko 24, LT-03225 Vilnius, Lithuania; urte.samukaite-bubniene@chf.vu.lt (U.S.-B.); vilma.ratautaite@ftmc.lt (V.R.); ieva.plikusiene@chgf.vu.lt (I.P.); roman.viter@lu.lv (R.V.); 3 Department of Mechatronics, Robotics, and Digital Manufacturing, Vilnius Gediminas Technical University, J. Basanaviciaus 28, LT-03224 Vilnius, Lithuania; inga.morkvenaite-vilkonciene@vgtu.lt; 4 Laboratory of Electrochemical Energy Conversion, State Research Institute Centre for Physical Sciences and Technology (FTMC), Sauletekio av. 3, LT-10257 Vilnius, Lithuania; 5 Laboratory of Nanotechnology, State Research Institute Center for Physical Sciences and Technology (FTMC), Sauletekio av. 3, LT-10257 Vilnius, Lithuania; 6 Institute of Atomic Physics and Spectroscopy, University of Latvia, Jelgavas Street 3, LV-1004 Riga, Latvia

**Keywords:** molecularly imprinted polymers (MIPs), biosensors, immunosensors, conducting polymers (CPs), conjugated polymers, polypyrrole, electrochemical deposition, polymer-modified electrodes, electrochemical sensors, electroanalysis

## Abstract

This review is dedicated to the development of molecularly imprinted polymers (MIPs) and the application of MIPs in sensor design. MIP-based biological recognition parts can replace receptors or antibodies, which are rather expensive. Conducting polymers show unique properties that are applicable in sensor design. Therefore, MIP-based conducting polymers, including polypyrrole, polythiophene, poly(3,4-ethylenedioxythiophene), polyaniline and ortho-phenylenediamine are frequently applied in sensor design. Some other materials that can be molecularly imprinted are also overviewed in this review. Among many imprintable materials conducting polymer, polypyrrole is one of the most suitable for molecular imprinting of various targets ranging from small organics up to rather large proteins. Some attention in this review is dedicated to overview methods applied to design MIP-based sensing structures. Some attention is dedicated to the physicochemical methods applied for the transduction of analytical signals. Expected new trends and horizons in the application of MIP-based structures are also discussed.

## 1. Introduction

In order to simplify analysis and to reduce costs, various affinity sensors are currently applied for various practical needs. In these sensors, a variety of analytical signal transduction methods are used to achieve sufficient sensitivity. However, the achievement of reliable selectivity is a much more complicated task; therefore, to resolve this challenge, many new and/or chemically advanced materials are designed [1,2]. Saliva, blood, blood serum, urine and other biological liquids contain some markers that are important for biomedical purposes. Currently, catalytic and affinity-based biosensors are most frequently used for the determination of some biologically active compounds that are present in biological samples [3,4].

To improve the selectivity of chemical sensors, many different semiconducting structures are designed [5,6]. It should be noted that, rather frequently, conducting polymers (CPs) are applied in the design of these sensing structures. Conducting polymers can be formed in many different ways; therefore, they are applied for the design of sensing structures, which increases the selectivity of the analytical method towards target compounds [1]. Conducting polymers are electrically conducting [7], have great electrical capacitance [8,9,10], can be well adhered to surface electrodes and form mechanically stable layers [11,12]. Some CPs demonstrate a great ability to transfer electrical charges and are used for electron transfer from some redox proteins and other biological structures [13]. Due to the above-mentioned properties, CPs are applied in the design of sensing layers used together with different signal transducers. In sensors and biosensors, the most frequently applied CPs are polypyrrole (Ppy), polyaniline (PANI), polythiophene (PTH) and poly(3,4-ethylenedioxythiophene) (PEDOT) [14,15,16,17,18]. Many different polymerization methods are used to design sensing structures based on conducting polymers, which are divided into chemical synthesis [19], electrochemical deposition [8], enzymatic formation [20] and/or microorganism assisted polymerization [21,22,23]. Moreover, CPs can serve as matrices for the immobilization of biomaterials, which are selectively binding targeted analytes, namely DNA [24], antibodies [1] receptors [25], antigens [26], antibodies [20] and enzymes [27,28,29]. These immobilized biomolecules provide specific selectivity to CP-based sensors. However, these biomolecules are not very stable and are very expensive; therefore, suitable replacements of these compounds are demanded. One of the most attractive alternatives to natural biological recognition compounds is based on the application of ‘synthetic receptors, mimics of antibodies and/or molecularly imprinted polymers (MIPs) [30,31], where conducting polymers are finding very great applicability [30,31,32,33,34,35]. In numerous researches, it was demonstrated that MIPs can be applied in the development of sensors for the diagnosis of infectious [36] and some other diseases.

Herein, we overview methods applied for the design of conducting polymer-based sensors and the modification of these polymers by molecular imprints of different molecules.

## 2. Chemical Formation of Conducting Polymers Based on Redox Processes

**Figure 1:**
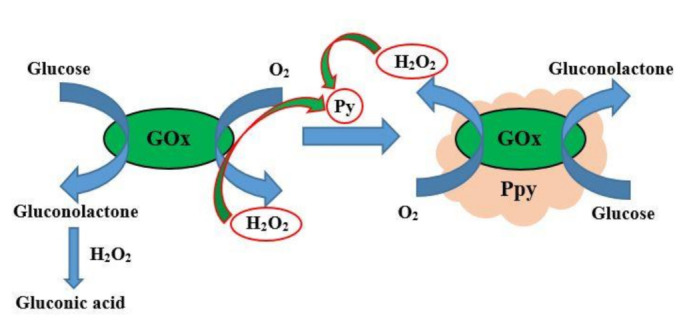
Chemical synthesis of polypyrrole, which was performed by glucose oxidase (GOx) assisted polymerization, where H_2_O_2_ is formed by GOx and acts as an initiator of the polymerization reaction. Figure adapted from [50].

The correct chemical formation is required for the development of conducting polymer-based nano- or/and micro-particles suitable for the design of molecularly imprinted polymer-based layers used for chemical sensors and chromatographic systems, and many other purposes [37,38]. Electroactive polymers are used not only for sensor design [39,40] but also for tissue regeneration and some other biomedical purposes [41]. Many approaches for the formation of conducting polymers are elaborated in order to fulfill technological demands. Chemical methods are very frequently used for the synthesis of large quantities of CPs, where polymerization is initiated by oxidators, namely FeCl_3_, H_2_O_2_ etc. [42,43,44]. The application of H_2_O_2_ enables the formation of very pure conducting polymers because the unreacted H_2_O_2_ rather quickly degrades into oxygen and water. Figure 1 represent the enzymatic synthesis of conducting polymer polypyrrole [19,44]. Many others conducting polymers, including polythiophene [44,45], poly-phenanthrenequinone [46], poly(pyrrole-2-carboxylic acid) [47], polyphenanthroline [13], azobenzene [48], carbazole [49], were designed using approaches similar to that represented in Figure 1.

In several of our researches, we have shown that some conducting polymers (e.g., polypyrrole) possess very good compatibility with stem cells and retain their biological activity [51,52]. A very important finding demonstrated by our research team illustrated that polypyrrole particles injected into the peritoneum of mice do not irritate the immune system of these animals [53]. Simple chemical polymerization procedures enable the synthesis of large quantities of various conducting polymers, which in addition can be additionally functionalized by entrapped molecules and other structures. Hence, various biomolecules, nanostructures, organic molecules, inorganic compounds and even ions can be incorporated within chemically formed conducting polymers [29,47,54,55].

Various redox enzymes, including oxidases, can be applied to perform the synthesis of conducting polymers. Glucose oxidase is the most frequently used for the formation of conducting polymers [20,29,47,54,55]. A very important fact is that such enzymatic synthesis can be pursued in an aqueous environment at ambient pH and temperature [56]. During the enzyme-assisted synthesis of conducting polymers, dissolved [54] and immobilized [27,29,47,55] enzymes (e.g.: glucose oxidase) can be used as producers of hydrogen peroxide and after theself-encapsulation within the formed conducting-polymer matrix these enzymes retain their catalytic activity. This method is well suited for the tuning of catalytic characteristics (especially for the adjustment of apparent Michaelis constant, which is mainly affected by the reduced diffusion rate of the substrate through a formed polymer layer) of enzymes immobilized in such conducting polymer-based structures. Such composite structures, which are based on immobilized enzymes, can be applied in the development of biosensors and biofuel cells [1,4,57,58].

It should be noted that conducting polymer (e.g., polypyrrole) formation can be induced by other oxidizing compounds, e.g., Fe^3+^ of [Fe(CN)_6_]^3−^ ions [59]. Therefore, metabolic redox reactions, which are running in microorganisms, can be involved in redox cycling of [Fe(CN)_6_]^3−^/[Fe(CN)_6_]^4−^ ions and in such a way can be exploited for the initiation of conducting polymer formation [21,22,23]. In some particular cases, nano-composite-based structures based on conducting polymers (e.g., PANI, Ppy, etc.) with embedded gold nanoparticles (AuNPs) and glucose oxidase (GOx) PANI/AuNPs-GOx can be designed [60]. Chemical polymerization is a rather basic procedure, which is suitable for the production of large quantities of MIPs [61,62]. Chemically formed conducting polymers can be deposited on transducer surfaces by spin coatings, solvent casting and other surface modification techniques [63]. However, most conducting polymers are not soluble in conventional solvents; therefore, technologically, it is not easy to deposit a sensing layer on the surface of the transducer. This challenge can be overcome using electrochemical conducting polymer formation methods.

## 3. Formation of Conducting Polymers by Electrochemical Methods

There are many methods used for the electrochemical formation of conducting polymers [64]; therefore, there is plenty of room to perform various modifications of CP-based layers during the deposition procedure. Characteristics of formed CP-based layers and other structures depend on parameters applied during the deposition procedure. The most important electrochemical deposition parameters are: (i) applied voltage, (ii) potential sweep rate used when potential cycling is applied, (iii) the duration of applied potential pulses and periods between them when potential pulse techniques are used, (iv) the control of charge, which is passing through the cell, [65,66], (v) the variation of ion and material concentrations that are present in the polymerization-bulk solution [67,68,69], (vi) treatment by ultrasound and some other external factors [70]. Thickness, density, ion permeability and many other characteristics of electrochemically deposited films are mostly affected by these conditions [26,71,72]. The porosity of the chemically sensitive layer, which is one of the most important factors affecting the analytical performance of the sensing layer, can also be changed by the aforementioned parameters [73,74,75]. Therefore, the adaptation of the above-listed parameters paves a way to design CP-modified electrodes with different electrochemical and analytical properties. In addition, the electrochemical modification of electrodes by conducting polymers can be instantly controlled by measuring/calculating the resistance and capacitance of the formed layer [26]. Polypyrrole [8,11,14,15,26,30,76], polyaniline [60], poly-9,10-phenanthrenequinone [46] and polythiophene derivatives [42], are among the most frequently electrochemically formed conducting polymers.

**Figure 2:**
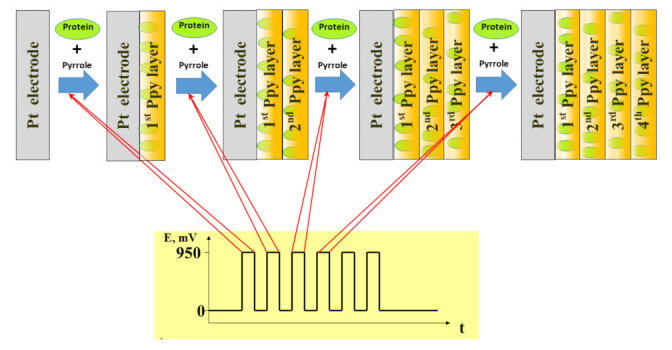
Electrochemical deposition of polypyrrole with simultaneously entrapped proteins using potential pulse-based technique, figure adapted from [50].

Various nanostructures (e.g., carbon nanotubes [77], molecules [35] and/or ions) can be entrapped within the formed conducting polymer layer, see Figure 2 [50]. It is very remarkable that these molecules and/or ions can be extracted from the polymer matrix using various procedures and in such a way that molecularly imprinted polymers are designed. Many polymerization approaches (including the above-mentioned electrochemical and chemical procedures) are used for the formation of MIPs. However, we believe that electrochemical deposition of MIP-based layers is more advantageous [78,79] when compared to chemical methods because electrochemical deposition provides much more options for variations in thickness, morphology and doping/de-doping of MIP-based structures. In addition, many other electrochemically performed procedures can be applied to advance these formed structures. One very useful procedure is overoxidation, which can be performed by the electrochemical treatment of film by positive electrode potentials that are much higher than those required for the initiation of a polymerization reaction [50]. Sometimes overoxidation occurs spontaneously when dissolved oxygen is present in the solution of polymerization-bulk and/or very active oxygen forms are electrochemically formed at the anode of the electrochemical cell.

It should be noted that, in some cases, overoxidation can terminate the polymerization reaction and/or destroy the conjugated π–π system, which is responsible for the good electrical conductivity of conducting polymers.

Overoxidation is very advantageous for the design of MIPs because groups (-COOH (carboxyl), >C=O (carbonyl), -OH (hydroxyl)), which are capable of hydrogen bond formation and the establishment of other electrostatic interactions, are formed and arranged in proximity with entrapped molecules. After the extraction of imprinted molecules, these carboxyl, carbonyl and hydroxyl groups form a recognition-able structure, which provides advanced selectivity towards the imprinted molecule. Therefore, overoxidation performed after the electrochemical formation of the conducting polymer layer is a very advantageous procedure. In some particular cases, overoxidation can help to remove imprinted molecules [80]. Overoxidized polypyrrole formed on a glassy carbon electrode was applied to detect Adefovir [81], and overoxidized polypyrrole formed on a glassy carbon electrode functionalized by a carboxylic acid multi-walled carbon nanotube layer was applied for the detection of pemetrexed [82].

Polypyrrole can be deposited electrochemically from pyrrole aqueous solutions by different electrochemical approaches [83] that can be easily controlled by computerized software [26]. Ppy-based MIPs were implemented in sensors dedicated for the detection of low molecular mass analytes such as: dopamine [84,85], theophylline [30,86], caffeine [14,15,87], histamine [88], quercetin [89], gallic acid [90], bilirubin [91], sarcosine [92], tetracycline [83], microcystin-LR [93], sulfanilamide [94], ganciclovir [95], uric acid [96], serotonin [97], L-aspartic acid [98], cysteine enantiomers [99], kanamycin [100], dibutyl phthalate [101], epinephrine (adrenaline) [102,103], coccidiostat clopidol [104], tryptamine [105], testosterone [106], fenvalerate [107] and NO^3−^ ions [108]. A summary of conducting polymer-based MIPs implemented in electrochemical sensors and dedicated to the detection of some low molecular mass analytes is listed in Table 1.

Phenylenediamine-derivatives [120,121,122] are also often used in the design of MIPs, which are applied for pharmaceutical and bioanalytical needs. Ortho-phenylenediamine was imprinted by anticancer drugs such as pemetrexed [123] and butyrylcholinesterase [124]. Electrochemically formed poly-meta-phenylenediamine MIP imprinted by erythromycin was used for the detection of erythromycin in real water-based aliquots [120]. Electrochemical dopamine sensors on poly-nicotinamide [125], sulphanilamide imprinted polyresorcinol [126], poly(1-naphthylamine), triphenylamine-based molecularly imprinted polythionine [127] or copolymer imprinted by azorubine [128] were previously reported. Recently, MIPs were used for the detection of some newly developed anticancer drugs [129].

Structures based on conducting polymers are environmentally stable and can be easily modified in many different ways [27]. Conducting polymers can be involved in the formation of composite structures modified by organic materials [130], inorganic structures [29,47,54,55] and biomolecules [44,54,60,131,132]. For this reason, they are often applied in the development of transducers for sensing and biosensing devices [133]. The doping of conducting polymers by particular ions and/or materials can significantly increase the electrical conductivity of conducting polymers [134].

## 4. MIPs Structures Imprinted by Biomolecules of High Molecular Weight

A variety of protein-based affinity sensors were designed via the immobilization of proteins within various polymer-based structures. These sensors are called immunosensors. The action of immunosensors is based on immobilized proteins that recognize analytes, whih are mostly other proteins or polypeptides, and form an immune complex, which induces the measurable signal of a particular signal transducer [135]. The orientation of immobilized protein molecules is also a key issue during the development of affinity sensors [25,136] because it determines the efficiency of analyte binding to the protein-modified surface [136]. Therefore, analytical characteristics of affinity sensors can be advanced by the proper orientation of immobilized receptors [25], whole antibodies [137], or fragments of chemically-split antibodies [136]. The electrochemical formation of conducting polymers is suitable for the entrapment of proteins in a formed polymer layer [26]. However, the successful application of molecular imprinting within polymers enables us to design MIPs that bind the imprinted analyte molecules [96,138,139].

**Figure 3:**
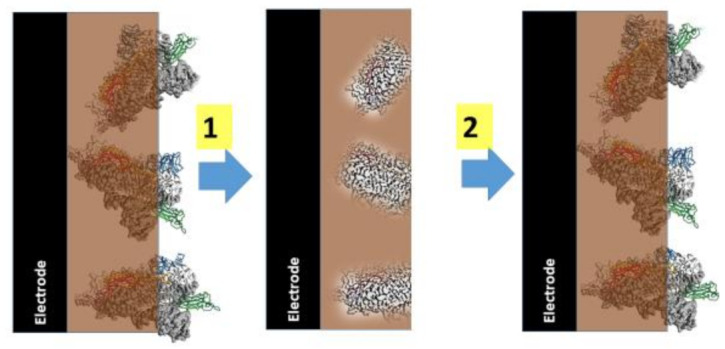
(**1**) Formation of molecularly imprinted polymer-based sensor, (**2**) MIP based layer in action. Figure adapted from [1].

The imprinting of polymers by proteins is a promising direction of MIP technology [140,141] (Figure 3) and can replace less stable proteins (such as antibodies [26] and receptors [25]), which are used in the design of affinity biosensors. It should be noted that the development of protein imprinted MIPs is a very promising direction of bioanalytical chemistry [142,143,144,145], some conformational variations of protein structure when protein is entrapped within a polymer [146], or proper protein orientation within polymers [147]. Molecularly imprinted polymers are sometimes called‘synthetic receptors’, ‘artificial receptors’ [143] or ‘plastic antibodies’ [148,149].

Bovine leukemia virus glycoprotein imprinted polypyrrole was applied in a MIP-based sensor design [143]. Electrochemically deposited poly o-phenylenediamine/hydroquinone imprinted by human serum albumin (HSA) was used for the detection of HSA in urine [150]. Polydopamine was surface-imprinted by immunoglobulin G [151]. Surface-imprinted PEDOT/PSS-based structures were also applied for the binding of proteins [147]. Synthetic receptors based on electrodeposited dopamine were applied for the detection of a prostate-specific antigen in samples of human blood plasma [152]. Electrochemically deposited polypyrrole/(carbon nanotube) composite was imprinted by S-ovalbumin and used for the determination of this albumin in egg white [153]. Electrochemically formed poly-o-phenylenediamine was imprinted by myoglobin [154]. Electrochemically formed MIP based on polyscopoletin was applied for the determination of HSA [155]. Poly-scopoletin was imprinted by cytochrome c [156]. A copolymer based on hydroxyethyl acrylate and ethylene glycol dimethacrylate imprinted by lysozyme was developed [157]. Poly(2-hydroxyethyl methacrylate-N-methacryloyl-(L)-histidin-Cu(II)) was formed using radical polymerization and imprinted by ceruloplasmin [158]. SARS-CoV-2 spike glycoprotein was imprinted into polypyrrole and deposited on a platinum electrode [159]. An electrochemical sensor based on poly-m-phenylenediamine imprinted by SARS-CoV-2 protein was applied for the diagnosis of COVID-19 [122]. Hexagonally packed macroporous molecularly imprinted polymers for chemosensing of follicle-stimulating hormone protein were developed [160].

Electrochemically deposited ortho-polydopamine was imprinted by alpha-fetoprotein, which before deposition of ortho-polydopamine was temporarily covalently immobilized on a gold nano-particle covered substrate [161]. Acrylamide/N,N0-methylenebisacrylamide copolymers were imprinted by two compounds: prostate-specific antigen and myoglobin [162]. Polyacrylamide imprinted by hemoglobin was designed [163]. O-phenylenediamine was used for the detection of imprinted troponin T that is an important biomarker for the diagnosis of early cardiac disease [164]. Conducting polymer polyaniline (PANI) is very often used in the design of sensors and biosensors [1], but despite the design of molecularly imprinted polymers, only very few research reports on PANI imprinted by antibiotic azithromycin [165] and by some saccharides and hydroxy acids exist [166]. It is remarkable that even titanium dioxide (TiO_2_) was imprinted by the enzyme urease, and it was applied in the sensor for the potentiometric determination of urea [167]. Very recently, it was demonstrated that the epitope imprinting approach applies exposed peptides as templates to synthesize electrochemically Molecularly Imprinted Polymers (MIPs) for the recognition of the parent protein [168]. Using this technology, epitope (N-terminal pentapeptide VHLTP-amide of human hemoglobin (HbA)) imprinted polymers were electrochemically deposited on an electrode surface.

MIP-formation is a highly multidisciplinary approach, which involves polymer and organic chemistry and nanotechnology [169,170]. It should be noted that DNA [27] can also be entrapped [24] or molecularly imprinted [171,172,173] within conducting polymers and applied for bioanalytical purposes. Therefore, a lot of researchers are targeted towards the replacement of biomolecules, such as receptors, antibodies, DNA-based sequences and DNA-aptamers, with molecularly imprinted polymer-based structures or by artificial receptors [174].

Even larger structures such as bacteria and other microorganisms [175,176] (e.g., bacteria *Escherichia coli* [177] or spores of *bacillus cereus* [178]) were imprinted within electrochemically deposited polypyrrole. Some more MIPs imprinted by viruses [179], spores [178], bacteria [177,180,181,182,183] and even some other living cells [184] were designed. Bacteria imprinted polymers can be used for the diagnosis of bacteria-induced infections and/or some bacterial toxin-based diseases [36,185].

## 5. Physicochemical Methods Used in Molecularly Imprinted Polymer Based Analytical Systems

MIP-based structures can selectively interact and bind various molecules. Moreover, in comparison to bio-receptors and enzymes, MIPs are better resistant to harmful environmental factors such as pH, temperature, etc. Some molecularly imprinted polymers are very stable at room conditions. Therefore, molecularly imprinted polymers seem very promising in the design of more stable and cheap sensing layers [186,187]. Due to the abovementioned reasons, the application of molecularly imprinted polymers in the design of affinity sensors is a rather new and promising direction of sensorics [188]. The highest stability is observed for MIPs based on acrylamide, methacrylic acid and acrylic acid [113,189,190,191,192].

The application of a suitable polymer for the design of a molecularly imprinted polymer plays a significant role in the design of efficient MIPs [193], which interact with analytes by the establishment of hydrogen bonds and π–π interactions, electrostatically, through van der Waals forces, and/or by hydrophobic-based interactions [194]. All these interactions are capable of dissociation; therefore, MIP-based sensors can be easily dissociated [2,191,195]. The formation and mechanism of action of molecularly imprinted polymers can be explained on the background of the phenomenological thermodynamic model, which explains the shape-memory effect of MIPs by solution theories derived by Flory and Huggins [196,197], Hansen [198] and Hildebrand [199]. Molecularly imprinted polymer shape changes are very often induced by swelling [200], which can significantly influence the ‘shape memory’ induced behaviour of these polymers [201]. In order to design molecularly imprinted polymers with desired analytical properties, molecular dynamics [202] and Density-Functional-Theory (DFT) [203,204] based calculations were recently performed.

**Figure 4:**
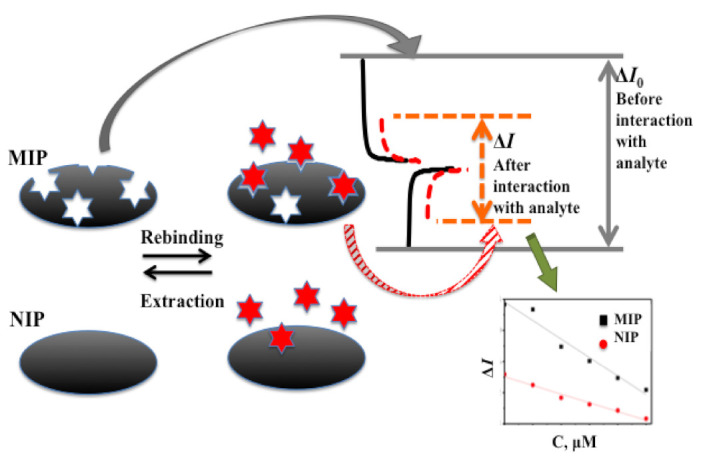
The principle of operation of molecularly imprinted and non-imprinted polymer-based sensors.

Therefore, electrochemical methods are very frequently used for the registration of analytical signals by sensors based on MIPs (Figure 4). Direct and indirect electrochemical detection methods are applied for the determination of analyte binding with MIPs. During direct measurements, the quantification of analyte binding to molecularly imprinted polymers is determined by different action explanations: (i) The most simple effect is derived from the so-called ‘gate effect’ and is based on rearrangements of MIP-based layer structures by swelling or shrinking that can be induced by analyte binding with an imprinted cavity. These variations of polymer structure affect the ion diffusion rate within the MIP-based layer and can be sensitively determined by a variety of potentiodynamic electrochemical methods [205,206]; (ii) Another effect is based on the charges of molecularly imprinted polymers, which are interacting oppositely charged ions and/or restrict the diffusion of some ions. Moreover, when analytes bind to molecularly imprinted polymers their electronic structure can be rearranged, which induces variations in the electrical conductivity of the sensing layer [205]. On the basis of the aforementioned effects, organic electrochemical transistors based on MIPs can be designed [207]. Dependent on doping conducting polymers, namely polyaniline (PANI) [208], polypyrrole (Ppy) [26] and poly(3,4-ethylenedioxythiophene)/poly(styrenesulfonate) (PEDOT/PSS) [209] are p- or n-type semiconductors. Doping and/or ‘un-doping’ of conducting polymers by some ions and materials is largely a reversible process responsible for the appearance of easily detectable variations of physical properties such as conductivity, light absorption or photoluminescence ability.

However, direct electrochemical methods have a significant disadvantage because all nonspecific interactions influence analytical signals. Therefore, in some particular cases, various redox-active probes are applied in order to improve the selectivity and sensitivity of MIP-based sensors [210,211]. In some cases, enzymatic activity is utilized for the enhancement of the analytical response in the design of such assays where tyrosinase [212], glucose oxidase [213], acetylcholinesterase [214], creatine kinase [215], cytochrome P450 [216], hexameric heme protein [217], laccase [218], microperoxidase [219], horseradish peroxidase [219,220,221], lactoperoxidase [219] are exploited. Sometimes enzyme-like activities, Pt/Cu bimetallic nanoparticles [222] and the inhibition of enzymatic activity [223] can be exploited amplifiy the analytical signal of MIP-based sensors.

**Figure 5:**
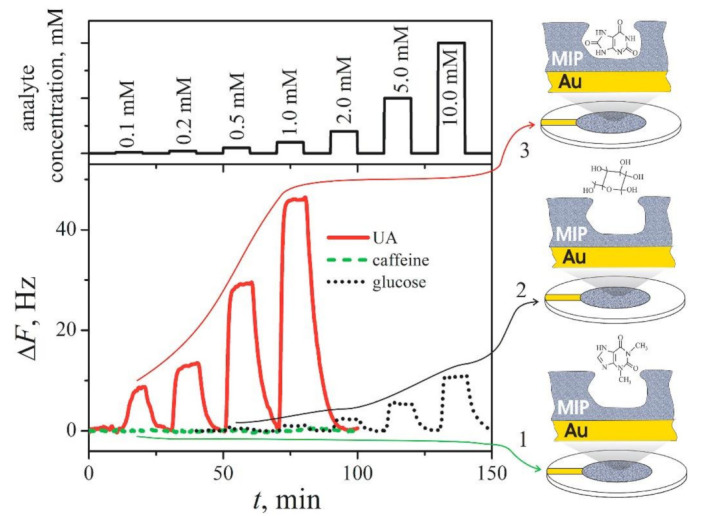
Variations of resonant frequency of an EQCM-resonator modified by uric acid-imprinted MIP(UA)-Ppy to the addition of different concentrations of uric acid (solid line 3), caffeine (dashed line 2) and glucose (dotted line 1) [96]. Copyright 2021 by Elsevier. Reprinted with permission.

Quartz crystal microbalance (QCM) with MIP-modified resonators [224] were used for the detection of: (i) some rather low molecular weight compounds [224,225], such as naproxen [226], histamine [227], *S*-propranolol [228], uric acid (Figure 5) [96], and ibuprofen [229]; (ii) proteins [176,230,231,232], such as trypsin [233], ribonuclease A [234] and oxidized-low-density lipoprotein [235]; and (iii) DNA [232,236]. QCM-based determination of the changes of the mass of the formed MIP layer can be simultaneously performed with electrochemical methods and can be used as an electrochemical-QCM (EQCM) method [15,96,237]. A more advantageous QCM-based technique, QCM with dissipation (QCM-D), was also used for the registration of analytical signal generated by MIP-modified resonators [238].

Various optical analytical signal registration methods, including photoluminescence [208,239] surface plasmon resonance (SPR) [240], can also be applied in MIP-based sensors. Remarkable optical characteristics of CPs can be well applied in the design of sensors based on optical transducers [241,242] and photoluminescence sensors [208,243,244]. We have demonstrated that conducting polymer polypyrrole has a great photoluminescence quenching ability [208,243], which can be exploited in the design of sensing devices and advance sensitivity and selectivity of biosensors [245]. Molecularly imprinted polymers can also be utilized for the detection of some organics, e.g., estradiol-derivatives [246,247,248]. MIP-based on quantum dot nanoparticles modified by poly(ethylene-co-vinyl alcohol) heterocomposite was used for optical detection of some salivary proteins [249]. MIP-based on a Cu^2+^-metalorganic-framework imprinted by tetrabromobisphenol A exhibited enzyme-like catalytic activity towards the oxidation of tetrabromobisphenol A by hydrogen peroxide [250]. Microarrays based on poly-scopoletin imprinted by ferritin were electro-spotted on a gold-modified substrate and applied in surface plasmon resonance (SPR)-based ferritin detection [251]. To advance optical capabilities, MIPs can be combined with photonic crystals [252] and liquid crystals [253]. A very useful optoelectrochemical property of some conducting polymers is their electrochromic effect, which can be exploited in the development of sensing devices.

## 6. Conclusions

MIPs are frequently used in the design of chemical sensors and biosensors, as well as for many other technological approaches. Some sensors based on MIPs provide fast analytical responses, operate at ambient conditions and are characterized by good sensitivity and selectivity. Some conducting polymers are well suited for the formation of MIPs, and these polymers can be designed using different polymerization methods. The electrochemical formation of CP-based structures can be controlled in many different ways and enables the ability to design of very different CP-based structures even from the same composition of polymerization-bulk solution; therefore, they are very well suited to the development of a great variety of MIPs. Some conducting polymers can be overoxidized after formation; this treatment is especially suited to the development of MIP-based sensors because it can be applied for (i) the formation of oxidized radicals, which increase sensitivity/selectivity towards imprinted target molecules, within MIP-based structures, (ii) the facilitation of template removal and/or regeneration of MIP-based layers.

Polypyrrole is the most used conducting polymer, and it is often applied in the formation of MIPs. Moreover, the advantages of the overoxidation of conducting polymer polypyrrole are the most frequently reported. However, in our opinion, this application of overoxidized polypyrrole still has a lot of room for improvement and extension in the application of polypyrrole based MIPs because polypyrrole can be easily synthesized by chemical and electrochemical methods from various solutions based on the most frequently used solvents. In addition, overoxidation of polypyrrole can be easily performed during the synthesis and/or after the formation of a Ppy-based layer. Moreover, polypyrrole shows great compatibility with various biological compounds and does not irritate the immune system of mammalians; therefore, it is suitable for the development of implantable biomedical tools, such as sensors, biosensors, biofuel cells, etc.

## Figures and Tables

**Table 1:** A summary of conducting polymer-based MIPs implemented in electrochemical sensors and dedicated to the detection of some low molecular mass analytes.

Analyte	Polymer and Modifiers of the Polymer	Method of Analysis	Analytical Parameters: Linear Range (LR), Limit of Detection (LOD)	Ref.
Neurotransmitters:				
Dopamine	*o*-phenylenediamine (OPD)-based MIP with polypyrrole nanowires on glassy carbon electrode	Differential pulse voltammetry (DPV)	LR from 50 nM to 0.1 mM; LOD 33 nM	[84]
	*o*-aminophenol (other monomers were bromophenol blue, picolinic acid, lactamic acid, and pyrrole) on nanoporous Au–Ag alloy microrod (NPAMR) as working electrode	Cyclic voltammetry (CV)	LR from 0.2 pM to 20 nM; LOD 76.3 fM	[85]
	Polypyrrole on S-MoSe_2_/NSG/Au nanocomposite on GCE	DPV	LR from 0.05 μM to 1000 μM; LOD 0.02 μM	[109]
	Polypyrrole on graphene quantum dots (GQDs)/TiO_2_ nanotubes (NTs) on Ti foil	Photoelectrochemical	LR from 0.05 μM to 12.5 μM; LOD 0.018 μM	[110]
	Polypyrrole on carboxyl-functionalized multi-walled carbon nanotubes (MWNTs-COOH) onto a glassy carbon electrode (GCE)	DPV	LR from 0.625 μM to 100 μM; LOD 60 nM	[111]
Serotonin	Graphene quantum dots on two dimensional hexagonal boron nitride on GCE	CV	LT from 1 pM to 0.1 nM; LOD 0.2 pM	[97]
	Acrylate-based MIP	EIS	LOD 3.2 nM	[112]
	5-hydroxy tryptophan (5-HTP) and acrylamide (AM) with carbon nanotubes on GCE	DPV and CV	LR from 5.4 nM to 1.8 μM; LOD 0.18 nM	[113]
Histamine	Polypyrrole on boron doped nanocrystalline diamond electrode	EIS		[88]
	Metacrylic acid-based MIP on carbon paste electrode	CV	LR from 0.1 nM to 7 nM and from 7 nM to 40 μM; LOD 74 nM	[114]
	Metacrylic acid-based MIP on interdigitated electrode	EIS	LR from 100 ppm to 500 ppm In seafood samples	[115]
	p-aminobenzene sulfonic acid (*p*-ABSA) based MIP on GCE with gold nanoparticles	DPV	LR from 1 μM to 40 μM and 40 μM to 107 μM; LOD 0.6 μM, In beer and wine	[116]
Epinephrine (adrenaline)	Polypyrrole-based MIP with silica nanoparticles and multiwalled carbon nanotubes on GCE	DPV	LR from 0.3 μM to 1 mM; LOD 30 nM	[104]
	Nicotinamide-based MIP with reduced graphene oxide on GCE	CV	LR from 0.015 μM to 40 μM; LOD 1.97 nM	[102]
Purine derivatives:				
Theophylline	Polypyrrole based MIP on boron-doped oxygen terminated nanocrystalline diamond as working electrode	EIS		[31]
	Polypyrrole based MIP on boron-doped oxygen terminated nanocrystalline diamond as working electrode	EIS		[86]
	Poly(pyrrole-*co*-pyrrole-3-carboxylic acid) based MIP with polystyrene colloidal particles on QCM sensor	QCM		[117]
Caffeine	Polypyrrole based MIP on the gold coated QCM sensor	QCM		[15]
	Acrylate based MIP on SPE (Screen Printed Electrode).	Heat-Transfer Method (HTM)	1 nM	[87]
Uric acid	Polypyrrole based MIP on Gold- coated quartz crystal resonator	EQCM		[96]
	Polydopamine based MIP with carbon-enwrapped nickel nanoparticles (Ni@BC) on GCE	DPV	LR from 0.01 μM to 30 μM, LOD 8 nM	[118]
Amino acids				
L-aspartic acid	Overoxidized polypyrrole based MIP on Gold- coated quartz crystal resonator	EQCM	-	[98]
Cysteine	Overoxidized polypyrrole based MIP on GCE with Au nanoparticles (AuNPs)	CV		[99]
Tryptophan	Chitosan based MIP on an acetylene black paste electrode	CV	LR from 0.01 μM to 4 μM, LOD 8.0 nM	[119]
Sarcosine	Poly-aminothiophenol (p-ATP) based MIP on screen-printed gold electrode	EIS	LR from 0.011 μM to 17.9 μM, LOD 8.5 nM	[92]
Other analytes:				
Quercetin	Polypyrrole based MIP on GCE with MIL-101 (Cr) and MoS_2_	DPV	LR from 0.1 μM to 10.5 μM and from 10.5 μM to 700 μM, LOD 0.06 μM	[89]
Gallic acid	Polypyrrole based MIP on gold electrode with Fe_3_O_4_@ZIF-67	DPV	LR from 6 pM to 600 pM, LOD 0.297 pM	[90]
Bilirubin	Polypyrrole based MIP on ITO electrode coated with TiO_2_	Photoelectrochemical	LR from 0.03 μM to 28 μM, LOD 1 nM	[91]
Testosterone	*O*-phenylenediamine (*o*-PD) based on GCE with graphene oxide (GO)	EIS	LR from 1 fM to 1 μm, LOD 0.4 fM	[106]

## Data Availability

Not applicable.
